# Supplementary material for: Empirical methods for controlling false positives and estimating confidence in ChIP-Seq peaks
Source: BMC Bioinformatics. 2008 Dec 5;9:523. doi: 10.1186/1471-2105-9-523 (PMC2628906; doi:10.1186/1471-2105-9-523)
Supplement: Additional File 1 — A variety of html documents from the USeq web site detailing the available applications, their best usage, output file type descriptions, command line menus, etc. [file 1471-2105-9-523-S1.zip › USeqUserGuides/JavaDoc.zip!/JavaDoc/cgar/beans/package-summary.html]

cgar.beans


|  |  |  |
| --- | --- | --- |
| **Overview** \| **Package** \| Class \| **Use** \| **Tree** \| **Deprecated** \| **Index** \| **Help** | |  |
| **PREV PACKAGE**   **NEXT PACKAGE** | **FRAMES**    **NO FRAMES**     **All Classes** |  |


---

## Package cgar.beans

| **Class Summary** | |
| --- | --- |
| **AnalysisBean** |  |
| **AnalysisGroupBean** |  |
| **AnalysisProtocolBean** | describes how the analysis was conducted, provide means to upload a flat file, use Analysis.protocolNotes to describe any deviations or particulars from uploaded file for a particular experiment. |
| **AnalysisTypeBean** |  |
| **GenomeBean** | Specific genome build/ release info, ie NCBI35.1 |
| **LabGroupBean** | Info related to a particular lab group. |
| **LoginBean** |  |
| **ProcessedDataBean** | Pointer to processed data file, where appropriate keep data broken down by chromosome. |
| **ProcessedDataTypeBean** |  |
| **RawDataBean** | Pointer to raw data file and id in GNomEx. |
| **SpeciesBean** | Name of a species from which multiple builds will be associated. |
| **UserBean** | User info, where possible this should come from GNomEx, use U ids/ passwords, when not available store md5 salted encrypted passwords, do not use db specific encryption function, use Nix's MD5Crypt.java class. |
| **UserTypeBean** | User type for defining access/ permissions: CGAR Administrator - no restrictions Group Super User - no restrictions on view and edit within the lab Group Member - view there own and other group items, only edit there own Group Guest - only group view privileges, no edit Anonomous - no edit, only view www A user type is specific for a particular lab. |
| **VisibilityBean** | VisibilityBean: who can see it: Owner (person who entered the analysis), Group Members, WWW. |

---


|  |  |  |
| --- | --- | --- |
| **Overview** \| **Package** \| Class \| **Use** \| **Tree** \| **Deprecated** \| **Index** \| **Help** | |  |
| **PREV PACKAGE**   **NEXT PACKAGE** | **FRAMES**    **NO FRAMES**     **All Classes** |  |


---
